# Cefuroxime Axetil (Ceftin^®^): A Brief Review

**DOI:** 10.1155/S1064744997000343

**Published:** 1997

**Authors:** Rachel Deanne Leder, Deborah Stier Carson

**Affiliations:** 1 Pharmacy Practice St., Louis College of Pharmacy St. Louis MO USA; 2 Department of Family Medicine 171 Ashley Avenue Medical University of South Carolina Charleston SC 29425 USA


Infectious Diseases in Obstetrics and Gynecology 5:211–214 (1997)
© 1997 Wiley-Liss, Inc.

### Cefuroxime Axetil (Ceftin®): A Brief Review

**Rachel Deanne Leder[1] and Deborah Stier Carson[2]***
[1]*Pharmacy Practice, St., Louis College of Pharmacy, St. Louis, MO*
[2]*Department of Family Medicine, Medical University of South Carolina, Charleston, SC*

KEY WORDS
cefuroxime axetil; antimicrobial; uncomplicated gonorrhea

Cefuroxime axetil (Ceftin®, Glaxo Wellcome, Research Triangle Park, NC) is the oral prodrug formulation of the injectable antibiotic cefuroxime sodium. It has essentially the same antibacterial activity as its parent moiety, making cefuroxime the only second-generation cephalosporin with both an intravenous and oral formulation.

### STRUCTURE AND DERIVATION

Cefuroxime axetil is the 1-acetoxyethyl ester of cefuroxime. The axetil salt renders the molecule more lipophilic, thus allowing enhanced oral absorption.[1] Once cefuroxime axetil reaches the intestinal mucosa and portal blood flow it rapidly undergoes de-esterification to yield the active parent compound cefuroxime.[1]

### MECHANISM OF ACTION

Cefuroxime axetil is a second-generation cephalosporin that contains the classic β-lactam ring structure. Bactericidal activity in vivo is resultant of its binding to essential target proteins, termed the penicillin-binding proteins, which are located in the bacterial cell wall. Inhibition of these proteins leads to bacterial cell wall elongation and leakage, thus the bacteria are unable to divide and mature.[2]

### PHARMACOKINETICS

Cefuroxime axetil is available as a tablet and a flavored suspension. Although the tablets have undergone three product reformulations in an attempt to standardize absorption, the bioavailability issues have been resolved with the currently marketed tablet. Cefuroxime axetil is converted to the active moiety, cefuroxime, in less than 3 min once absorbed.[1] Due to the rapid conversion it is not possible to detect cefuroxime axetil in the systemic circulation.[1] Peak serum concentration achieved after a single 250 mg dose in the fed state is 4.7 mcg/ml and is reached after 2.1 h post-ingestion.[3] The administration of food with cefuroxime axetil substantially increases its absorption.[1,4,5] The bioavailability was shown to increase from 36% to 52% when a 500 mg dose was taken in a fasting state compared to being administered after food.[5] The mechanism for this increased bioavailability is not completely understood. It has been proposed that food-induced cholecystokinin release which causes the gall bladder to contract and release bile may be responsible for improving absorption.[6]

Cefuroxime axetil, as cefuroxime, is approximately 30% protein bound and has a volume of distribution of about 17 l.[7] Distribution of this antibiotic into body fluids and tissues is variable, however, it does penetrate well (35–90%) into the tonsil tissue, sinus tissue, and bronchial mucosa.[8]

Once de-esterified and released into systemic circulation, cefuroxime is not metabolized further, but is eliminated unchanged in the urine. In patients with normal renal function, the plasma elimination half-life after a dose of 500 mg of cefuroxime is 1.4. h. The elimination half-life increases as the renal function declines. In patients with creatinine clearances <10 ml/min the elimination half-life extends to approximately 16.8 h.[9] Based on these results, it is recommended that the dosing interval be

*Correspondence to: Dr. Deborah Stier Carson, Department of Family Medicine, 171 Ashley Avenue, Medical University of South Carolina, Charleston, SC 29425.

Received 1 April 1997

Accepted 23 June 1997

TABLE 1. Dosage guidelines for renal dysfunction[a]

| Estimated creatinine clearance | Recommended dosage |
|---|---|
| 30–49 ml/min/1.73 $m^2$ | Standard individual dose given every 12 h |
| 10–29 ml/min/1.73 $m^2$ | Standard individual dose given every 24 h |
| <10 ml/min/1.73 $m^2$ | Standard individual dose given every 48 h |

[a]Adapted from Konishi et al.[9]

extended in patients with renal dysfunction (see Table 1).

### SIDE EFFECTS AND INTERACTIONS

Cefuroxime axetil is associated with a low incidence of adverse effects and is generally well tolerated. The most frequently reported adverse effects are primarily gastrointestinal in nature, including diarrhea/loose stools (3.7%), nausea (2.6%), and vomiting (2.6%).[10,11] In clinical trials comprised of large cohorts of patients (n = 912) using multiple doses of cefuroxime axetil, only 2.2% of patients discontinued treatment due to adverse reactions. Of those who discontinued treatment, 85% did so because of gastrointestinal complaints.[10]

Other occasionally reported adverse events associated with cefuroxime axetil include antibiotic-associated colitis and liver function abnormalities. Hypersensitivity reactions including Stevens-Johnson syndrome, erythema multiforme, and toxic epidermal necrolysis have been reported rarely during post-marketing surveillance.[10,11]

Concurrent use of cefuroxime with probenecid may increase the serum concentration of cefuroxime. Use with warfarin may increase the hypoprothrombotic effect of the anticoagulant; therefore, closer monitoring of the patient's international normalized ratio during cefuroxime therapy is recommended.[10]

### SPECTRUM OF ANTIMICROBIAL ACTIVITY

Cefuroxime axetil has a wide spectrum of bactericidal activity both in vivo and in vitro against many gram-positive bacteria, some gram-negative bacteria, and few anaerobic bacteria. It even covers those strains that produce β-lactamases.[12] It is highly effective against many of the common respiratory pathogens including *Streptococcus pneumoniae, Haemophilus influenzae,* and *Moraxella catarrhalis.* It is one of the few oral cephalosporins with some activity against isolates of *S. pneumoniae* that are intermediately resistant to penicillin.[13] It also has good activity against the pathogens that lead to skin and soft tissue infections, including methicillin-sensitive staphylococci and *S. pyogenes.* The most frequently encountered urinary tract infection pathogens, *Escherichia coli, Proteus mirabilis,* and *Klebsiella pneumoniae,* are also susceptible to cefuroxime axetil. Cefuroxime axetil has coverage for both the penicillinase-positive and -negative strains of *Neisseria gonorrhea,* and thus is an alternative choice for uncomplicated gonorrhea. It has activity against *Borrelia burgdorferi,* the bacteria responsible for Lyme disease, and some anaerobic bacteria such as *Peptococcus* species. Most strains of *Clostridium difficile* and *Bacteroides fragilis* are resistant, and therefore render this antibiotic a poor choice for most obstetric and gynecological surgeries.[10,14]

*Pseudomonas* species, *Campylobacter* species, *Acinetobacter calcoaceticus,* most strains of *Serratia* species, *Proteus vulgaris,* and certain strains of *Enterococci* are resistant to therapy with cefuroxime axetil.[10] Resistance is being reported for some nosocomial isolates of Enterobacteriaceae, primarily *K. pneumoniae* and *E. coli.* This is due to bacterial production of novel plasmid-mediated β-lactamases.[15]

### CLINICAL APPLICATIONS

Multiple clinical trials have investigated the therapeutic efficacy of cefuroxime axetil in upper and lower respiratory tract infections, uncomplicated urinary tract infections, skin and soft tissue infections, uncomplicated gonorrhea, and early stage Lyme disease. Although cefuroxime axetil has labeled indications for all of the previously mentioned infections,[10] it is generally not the preferred antibiotic for initial treatment since equally efficacious and less expensive options are available. For general use in obstetrics and gynecological infections, cefuroxime axetil may be considered a useful alternative for treating uncomplicated gonorrhea and urinary tract infections (UTIs). It is considered safe to use in pregnancy (pregnancy category B).[16]

### Uncomplicated Gonorrhea

For treatment of uncomplicated gonorrhea, the Centers for Disease Control and Prevention (CDC) recommends ceftriaxone 125 mg IM once plus a 7

day course of doxycycline for the presumptive concurrent infection with *Chlamydia*. This regimen has a greater than 95% cure rate for anal and genital infections while also achieving cure rates of ≥90% for pharyngeal infections. Another advantage for ceftriaxone is it may also abort incubating syphilis, a concern when treatment is not accompanied by a 7 day course of doxycycline. Cefuroxime axetil 1 g orally as a single dose is considered an alternative regimen. However, a single dose of this shorter-acting cephalosporin will not cover incubating syphilis nor *C. trachomatis*.[17] Clinical trials conducted using 1–1.5 g single doses of cefuroxime axetil either alone or in combination with probenecid have produced cure rates of 96–100% in gonococcal genitorectal infections in both men and women.[18–24] However, these high cure rates were not achieved in patients with pharyngeal gonococcal infections.[20] In comparative trials vs. amoxicillin plus probenecid, cefuroxime axetil was shown to be equally effective.[19–21] There have been no comparative trials between cefuroxime axetil and ceftriaxone.

### UTIs

The most commonly identified UTI pathogens include *E. coli*, *K. pneumoniae*, and *P. mirabilis*. Cefuroxime axetil has been shown to be effective against these urinary pathogens, but provides broader coverage than necessary for most uncomplicated UTIs. The antibiotic of choice for the treatment of acute uncomplicated UTIs is trimethoprim in combination with sulfamethoxazole (TMP/SMX) or amoxicillin. For patients with an allergy to these agents, cefuroxime axetil is an expensive alternative. Several dosing regimens for treatment of UTIs have proved effective with cefuroxime axetil. In one clinical trial in non-pregnant women, single dose therapy with 1,000 mg resulted in a 88% clinical and bacteriological cure at 1 week post-therapy.[25] A slightly longer 3 day therapy trial with 125 mg twice daily had a similar efficacy, an 84.8% clinical cure.[26] The more traditional 7 day therapy 125 mg twice daily resulted in a 97% bacteriological cure at 1 week post-therapy.[27]

### COST

Table 2 represents the average wholesale price to the pharmacist for a 7 day supply of selected antibiotics. These selected antibiotics represent either first-line choices for therapeutic indications such as gonorrhea, UTI, and skin/soft tissue infection, or in the case of cefaclor, have a similar spectrum of activity. In most cases, cefuroxime axetil is the more expensive choice with no increase in efficacy or safety.

**TABLE 2. Comparison of cost of commonly prescribed antibiotics used to treat obstetric and gynecological infections**

| Drug | Daily dosage[a] | AWP cost for 7 day supply[b] |
|---|---|---|
| Amoxicillin | | |
| Generic | 250 mg q 8 h | $4.41 |
| Amoxil® | | $4.54 |
| Clavulanate potassium | | |
| –Augmentin® | 500 mg q 12 h | $40.32 |
| Cefaclor | | |
| Generic | 250 mg q 8 h | $40.90 |
| Ceclor® | | $46.49 |
| Cefuroxime axetil–Ceftin® | 250 mg q 12 h | $44.94 |
| | 1 g × 1 dose | $12.84 |
| Ceftriaxone–Rocephin® | 125 mg IM × 1 | $12.04 |
| Doxycycline–generic | 100 mg q 12 h | $2.24 |
| Trimethoprim-sulfamethoxazole | | |
| Generic double-strength | 1 tablet q 12 h | $4.76 |
| Bactrim DS® | | $17.50 |
| Septra DS® | | $16.94 |

[a]Usual adult dosage recommended by the manufacturer.
[b]Cost to the pharmacist based on average wholesale price (AWP) listings in *Drug Topics Red Book* (1996); cost to the patient may be higher.

### CONCLUSIONS

Cefuroxime axetil is a broad spectrum β-lactam antibiotic. It has many approved indications, however, it is considered a second-line alternative. It is not the drug of choice for any infection, particularly those encountered in the field of obstetrics and gynecology. It is safe to use in pregnancy and has a low adverse effect profile, but due to its excessive acquisition cost and better therapeutic alternatives, it should be reserved for select cases.

### REFERENCES

1. Harding MS, Williams PO, Ayrton J: Pharmacology of cefuroxime as the 1-acetoxyethyl ester in volunteers. Antimicrob Agents Chemother 25:78–82, 1984.
2. Mandell GL, Petri WA: Antimicrobial agents: Penicillin, cephalosporins, and other β-lactam antibiotics. In Hardman JG, Limbird LE (eds): Goodman and Gilman's The Pharmacological Basis of Therapeutics. 9th ed. New York: McGraw-Hill, pp 1074–1076, 1996.
3. Kees F, Lukassek U, Naber KG, Grobecker H: Comparative investigations on the bioavailability of cefuroxime axetil. Arzneim Forsch 41:843–846, 1991.
4. Ginsburg CM, McCracken GH, Petruska M, Olson K: Pharmacokinetics and bactericidal activity of cefuroxime axetil. Antimicrob Agents Chemother 28:504–507, 1985.
5. Finn A, Straughn A, Meyer M, Chubb J: Effect of dose and food on the bioavailability of cefuroxime axetil. Biopharm Drug Dispos 8:519–526, 1987.
6. Mackay J, Mackie AE, Palmer JL, et al.: Investigations into the mechanism for the improved oral systemic bioavailability of cefuroxime from cefuroxime axetil when taken after food. Br J Clin Pharm 33:226P–227P, 1992.
7. Foord RD: Cefuroxime: Human pharmacokinetics. Antimicrob Agents Chemother 9:741–747, 1976.
8. Perry CM, Brogden RN: Cefuroxime axetil—A review of its antibacterial activity, pharmacokinetic properties and therapeutic efficacy. Drugs 52:125–158, 1996.
9. Konishi K, Suzuki H, Hayashi M, Saruta T: Pharmacokinetics of cefuroxime axetil in patients with normal and impaired renal function. J Antimicrob Chemother 31: 413–420, 1993.
10. Glaxo Wellcome, Inc.: Cefuroxime Axetil. Package Insert. Research Triangle Park, NC: Glaxo Wellcome, Inc., 1995.
11. McEvoy GK, Litvak K, Welsh OH (eds): Cefuroxime axetil (monograph). In: American Hospital Formulary Service 1997 Drug Information. Bethesda, MD: American Society of Health System Pharmacist, pp 173–181, 1997.
12. Neu HC, Fu KP: Cefuroxime, a β-lactamase resistance cephalosporin with a broad spectrum of gram positive and negative activity. Antimicrob Agents Chemother 13: 657–664, 1978.
13. Bradley JS, Kaplan SL, Klugman KP, Leggiadro RJ: Consensus: Management of infections in children caused by *Streptococcus pneumoniae* with decreased susceptibility to penicillin. Pediatr Infect Dis J 14:1037–1041, 1995.
14. Sweet RL, Gibbs RS: Antimicrobial agents. In: Infectious Diseases of Female Genital Tract. 3rd ed. Baltimore: Williams & Wilkins, pp 680–683, 1995.
15. Sirot J, Chanal C, Petit A, et al.: *Klebsiella pneumoniae* and other enterobacteriaceae producing novel plasmid-mediated β-lactamases markedly active against 3rd generation cephalosporins: Epidemiological studies. Rev Infect Dis 10:850–859, 1988.
16. Briggs GG, Freeman RK, Yaffe SJ (eds): Cefuroxime (monograph). In: Drugs in Pregnancy and Lactation. 4th ed. Baltimore: Williams & Wilkins, pp 147–148, 1994.
17. Centers for Disease Control and Prevention: 1993 sexually transmitted disease treatment guidelines. MMWR 43(RR-14):57–59, 1993.
18. Gottlieb A, Mills J: Cefuroxime axetil for treatment of uncomplicated gonorrhea. Antimicrob Agents Chemother 30:333–334, 1986.
19. Reichman RC, Nolte FS, Wolinsky SM, et al.: Single-dose cefuroxime axetil in the treatment of uncomplicated gonorrhea: A controlled trial. Sex Transm Dis 12: 184–187, 1985.
20. Baddour LM, Gibbs RS, Mertz G, et al.: Clinical comparison of single-oral-dose cefuroxime axetil and amoxicillin with probenecid for uncomplicated gonococcal infections in women. Antimicrob Agents Chemother 33: 801–804, 1989.
21. Fong IW, Linton W, Simbul M, Hinton NA: Comparative clinical efficacy of single oral doses of cefuroxime axetil and amoxicillin in uncomplicated gonococcal infections. Antimicrob Agents Chemother 30:321–322, 1986.
22. Wanas TM, Williams PE: Oral cefuroxime axetil compared with oral ampicillin in treating acute uncomplicated gonorrhoea. Genitourin Med 62:221–223, 1986.
23. Das RP, Jones K, Robinson AJ, Timmins DJ: Cefuroxime axetil to treat gonorrhoea (letter). Genitourin Med 64:394, 1988.
24. Schift R, Van-Ulsen J, Ansink-Schipper MC, et al.: Comparison of oral treatment of uncomplicated urogenital and rectal gonorrhoea with cefuroxime axetil ester or clavulanic acid potentiated amoxicillin (Augmentin). Genitourin Med 62:313–317, 1986.
25. Iravani A, Richard GA: Single-dose cefuroxime axetil versus multiple-dose cefaclor in the treatment of acute urinary tract infections. Antimicrob Agents Chemother 33:1212–1216, 1989.
26. Naber KG, Koch EMW: Cefuroxime axetil versus ofloxacin for short-term therapy of acute uncomplicated lower urinary tract infections in women. Infection 21: 34–39, 1993.
27. Cooper J, Raeburn A, Brumfitt W, Hamilton-Miller JMT: Comparative efficacy and tolerability of cephradine and cefuroxime axetil in the treatment of acute dysuria and/or frequency in general practice. Br J Clin Pract 46:24–27, 1992.
